# The tomato NAC transcription factor *SlNAM2* is involved in flower-boundary morphogenesis

**DOI:** 10.1093/jxb/ert324

**Published:** 2013-10-01

**Authors:** Anat Hendelman, Ran Stav, Hanita Zemach, Tzahi Arazi

**Affiliations:** ^1^Institute of Plant Sciences, Agricultural Research Organization, Volcani Center, PO Box 6, Bet Dagan 50250, Israel; ^2^Department of Life Sciences, Bar Ilan University, Ramat Gan 52900, Israel

**Keywords:** Boundary, development, flower, miR164, NAC, tomato.

## Abstract

Being composed of several whorls of distinct floral organs, the flower is one of the most complex organs in the plant. As such, the formation and maintenance of boundaries that separate the meristem from the floral organ primordium and adjacent organs are critical for its normal development. In *Arabidopsis*, the miR164-regulated *NAM* genes play key roles in floral-boundary specification. By contrast, much less is known about floral-boundary establishment in the model crop tomato. It was found that the miR164-regulated *NAM* gene *GOBLET* is expressed in the floral meristem–organ boundaries and its loss-of-function mutant produces flowers with fused organs, indicating its requirement for tomato floral-boundary formation. It was found here that sly-miR164 targets the transcripts of three additional uncharacterized *NAM* genes in developing flowers. It is shown that, after floral-boundary initiation, the *NAM* gene *Solyc03g115850* (*SlNAM2*) is expressed as stripes that mark the boundaries between sepals and between different floral whorls. Furthermore, ectopic accumulation of SlNAM2-encoding transcripts caused various growth-suppression and extraorgan phenotypes typically observed in plants over-expressing known boundary genes. Flower-specific silencing of sly-miR164-targeted *NAM* genes (*AP1>>MIR164*) caused defects in the separation of sepals and floral whorls indicating abnormal boundary specification. However, supplementing these *NAM*-deficient flowers with miR164*-*resistant *SlNAM2* suppressed their fusion phenotypes and completely restored floral boundaries. Together, our results strongly suggest that *SlNAM2* participates in the establishment of tomato flower whorl and sepal boundaries.

## Introduction

The production of plant lateral organs depends on the formation of a narrow domain of non-dividing cells or boundary that separates the organ primordium from the meristem. In addition, normal lateral organ architecture requires proper organ–organ boundary formation that separates distinct tissues (Breuil-Broyer *et al.*, 2004; Aida and Tasaka, 2006*b*; Rast and Simon, 2008). It was found that the formation of boundaries is regulated by specific boundary genes that are expressed in the cells that will form the boundary. Accordingly, misexpression of these genes can lead to growth arrest or abnormal development (Aida *et al.*, 1997; Brewer *et al.*, 2004; Takeda *et al.*, 2004; Aida and Tasaka, 2006*b*). In *Arabidopsis thaliana*, several gene families are involved in the specification of meristem–organ and organ–organ boundaries. Prominent among them is the large family of plant-specific NAM, ATAF1/2, CUC2 (NAC) transcription factors which include several genes that participate in the establishment of meristem–organ and organ–organ boundaries (Ishida *et al.*, 2000; Takada *et al.*, 2001; Aida and Tasaka, 2006*b*; Raman *et al.*, 2008). A typical NAC transcription factor contains a highly conserved N-terminal DNA-binding NAC domain and a variable C-terminal region (Ooka *et al.*, 2003). Alignment of the C-terminal regions of closely related NAC proteins identifies common sequence motifs that correlated with their functionality. For example, the motifs LPPLxD and [E/x][H/x]VxCFS[N/x] have been found in most *Arabidopsis* and rice NAC proteins which belong to the NAM subclade and are involved in developmental plans (Ooka *et al.*, 2003). In addition to boundary morphogenesis, NAC genes are involved in various developmental processes such as root formation (Xie *et al.*, 2000), leaf senescence (Kim *et al.*, 2009), and biotic and abiotic stress responses (Delessert *et al.*, 2005; He *et al.*, 2005).

The *Arabidopsis CUP SHAPED COTYLEDON1* (*CUC1*) and *CUC2* are functionally redundant miR164-regulated *NAM* genes that promote boundary formation and maintenance throughout vegetative and reproductive development (Aida *et al.*, 1997; Takada *et al.*, 2001). Accordingly, they are expressed between the meristem and the new lateral organ, at the base of the outgrowing teeth defining the leaf sinuses, between the inflorescence meristem and the new flower meristem, and between floral-organ primordia (Ishida *et al.*, 2000; Takada *et al.*, 2001; Vroemen *et al.*, 2003; Nikovics *et al.*, 2006). Consistent with their requirement for boundary formation, *cuc1 cuc2* double mutants develop fused cotyledons and produce flowers with fused sepals and stamens and with fewer petals (Aida *et al.*, 1997). Similar phenotypes have also been observed in plants over-expressing miR164 (Laufs *et al.*, 2004; Mallory *et al.*, 2004). Gain-of-function of either *CUC1* or *CUC2* leads to extrafloral organ formation and variable growth-suppression phenotypes, which have been suggested to be caused by repression of cell division (Takada *et al.*, 2001; Laufs *et al.*, 2004; Mallory *et al.*, 2004; Baker *et al.*, 2005; Nikovics *et al.*, 2006; Sieber *et al.*, 2007).

To date, only a single *NAM* gene involved in boundary formation has been functionally characterized in tomato. A loss-of-function mutant of this gene produced goblet-shaped fused cotyledons and, accordingly, it was named *GOBLET* (*GOB*). Similar to its *CUC2* homologue, the spatial and temporal expression of *GOB* is post-transcriptionally regulated by sly-miR164. This regulation limits *GOB* expression to the boundaries between the shoot apical meristem and leaf primordia and between leaflet primordia (Blein *et al.*, 2008; Berger *et al.*, 2009). Accordingly, a loss-of-function *gob-3* mutant produced simpler leaves with smooth leaflet margins lacking secondary leaflets, and gain-of-function *Gob-4d* produced extralobed cotyledons and deeply lobed leaves, together indicating that *GOB* is required for the formation of the boundaries between leaflets in compound tomato leaves (Berger *et al.*, 2009). In the flower, *GOB* is expressed at the boundaries between floral meristem and floral-organ primordia (Blein *et al.*, 2008). In addition, *gob-3* and *Gob-4d* mutants produced flowers with fused sepals and fewer locules or with extra carpals, respectively, together suggesting that *GOB* functions in the formation of floral-organ boundaries as well (Berger *et al.*, 2009).

In the current study, *SlNAM2*, a new *NAM* gene that is post-transcriptionally regulated by sly-miR164, is functionally characterized and evidence is provided for its involvement in the establishment of floral boundaries.

## Materials and methods

### Plant material and growth conditions

Tomato (*Solanum lycopersicum*) cv. M82 lines *35S:LhG4* (Lifschitz *et al.*, 2006), *AP1:LhG4* (Fernandez *et al.*, 2009), and *OP:MIR164* (Alvarez *et al.*, 2006) have been described elsewhere. The tomato plants were grown under greenhouse conditions with temperatures ranging between 15 °Cand 25 °C in a tuff–peat mix with nutrients, using 4l pots. Germination and seedling growth took place in a growth chamber with a 16/8h light/dark period (photosynthetic photon flux density: 50–70 µmol m^–2^ s^–1^) at a constant temperature of 24 °C. For crosses, closed flowers were emasculated by removing the petals and stamens and hand-pollinated with the pollen of an appropriate homozygous driver line.

### Total RNA extraction and small-RNA blot analyses

Total RNA was isolated from different tomato tissues with Bio-TRI RNA reagent (Bio-Lab, Jerusalem, Israel) according to the manufacturer’s protocol. After the addition of isopropanol, the RNA extract was incubated overnight at –20 °C to enhance the precipitation of low-molecular-weight RNAs. Following an ethanol wash, RNA was resuspended in RNase-free water and kept at –80 °C until use. Small-RNA gel-blot analysis of 5 µg total RNA was performed as described previously by Talmor-Neiman *et al.* (2006). For the detection of sly-miR164 and *U6* small nucleolar RNA, a radiolabelled oligo probe that is complementary to the corresponding small RNA was used. For the detection of the *SlNAM2* small interfering RNAs (siRNAs), a 353bp fragment from the 3’ untranslated region (UTR) of *SlNAM2* was amplified by RT-PCR from tomato flower cDNA with the primers SLNAM2IR_ClaI-PstI_fwd and SlNAM2IR_HindIII-EcoRI_rev (all primer sequences are given in Supplementary Table S1 at *JXB* online) and then cloned into pGEM-T Easy (Promega, Madison, WI, USA) so that it was in the antisense orientation relative to the T7 promoter. Then a radiolabelled RNA probe was transcribed using the RiboScribe T7 probe synthesis kit (Epicentre Biotechnologies, Madison, WI, USA) in the presence of [α-^32^P]UTP.

### Target prediction and validation by cleavage-site mapping

Sly-miR164 targets were predicted by psRNATarget (http://plantgrn.noble.org/psRNATarget/, last accessed 09 September 13) (Dai and Zhao, 2011) against the current version of the publicly available genome [SGN ITAG release 2.3 predicted cDNA (SL2.40)]. For target validation, total RNA was extracted from tomato flowers as described above, and enriched for poly(A) mRNA using the Oligotex mRNA Mini Kit (Qiagen, Valencia, CA, USA). A modified procedure for RNA ligase-mediated rapid amplification of cDNA ends (5’ RLM-RACE) was performed with the GeneRacer Kit (Invitrogen, Carlsbad, CA, USA) as described previously by Talmor-Neiman *et al.* (2006). Briefly, cDNA was amplified with the GeneRacer-5’ primer and with SlNAM2_RACE, SlNAM3_RACE, SlNAC1_RACE, and GOBLET_RACE primers, followed by nested PCR using GeneRacer-5’-nested primer and SlNAM2_RACE_nested, SlNAM3_RACE_nested, SlNAC1_RACE_nested, and GOBLET_RACE_nested primers, respectively. The PCR conditions used for both amplification steps were as recommended by the manufacturer. The amplified products were gel-purified, cloned into pGEMT-easy vector (Promega) and sequenced. For transgenic *SlNAM2*, total RNA was extracted from young tomato leaves as described above, and 6 µg DNA-free total RNA was used to produce the RLM-RACE cDNA. The cDNA was subjected to an amplification procedure with the GeneRacer-5’ primer and the transgene-specific primer OCS_rev followed by nested PCR with primer pair GeneRacer-5’-nested and SlNAM2_RACE_nested. Amplification of the intact transgenic transcript was performed by RT-PCR with the primer SlNAM2_Exon_557_fwd found upstream of the sly-miR164 cleavage site and OCS_rev.

### Plasmids construction

For the *SlNAM2* reporter construct, the coding region of *SlNAM2* was cloned by RT-PCR from the flower cDNA with the primers XhoI-U218896_fwd and BamHI-U218896_rev, which contained *Xho*I and *Bam*HI sites at their 5’ ends, respectively. The amplified fragment was restricted with *Xho*I/*Bam*HI and cloned into the respective sites of the OP-TATA-BJ36 shuttle vector between an *OP* array (Moore *et al.*, 1998) and *Agrobacterium tumefaciens* octopine synthase terminator (OCS) to generate OP:SlNAM2. To generate OP:mSlNAM2, six silent mutations in the *SlNAM2* sly-miR164 target site were inserted using two-step PCR mutagenesis. Firstly, the 164-mutant-target_fwd and 164-mutant-target_rev primers were used in conjunction with BamHI-U218896_rev and XhoI-U218896_fwd, respectively, to insert six substitutions (lowercase letters in Supplementary Table S1 at *JXB* online) into the *SlNAM2*-coding region. Then, the amplified products were assembled by using them as a template for PCR with the primer pair XhoI-U218896_fwd and BamHI-U218896_rev. The amplified fragment was restricted with *Bam*HI/*Xho*I and cloned into the identical sites of OP:SlNAM2, replacing the respective wild-type *SlNAM2* fragment to generate OP:mSlNAM2. Following sequence validation, the *Not*I fragments of OP:SlNAM2 and OP:mSlNAM2 were mobilized into the pART27 binary vector (Gleave, 1992) to generate pART27-OP:SlNAM2 and pART27-OP:mSlNAM2, respectively. For the SlNAM2 RNA interference (RNAi) reporter construct, a 353bp fragment from the 3’ UTR of *SlNAM2* was cloned by RT-PCR from the flower cDNA with the primers SLNAM2IR_ClaI-PstI_fwd and SlNAM2IR_HindIII-EcoRI_rev, each containing two indicated restriction sites at their 5’ end. The amplified fragment was restricted with either *Pst*I/*Eco*RI or *Cla*I/*Hin*dIII and cloned in the sense and antisense orientations, respectively, around the first intron of the *Arabidopsis AKT1* gene to generate max2intpFLAP-SlNAM2IR. Following sequence validation, the *Xho*I fragment of the max2intpFLAP-SlNAM2IR was mobilized into the *Xho*I site of the OP-TATA-BJ36 shuttle vector to generate OP:SlNAM2IR. Following orientation validation, the *Not*I fragment of the OP:SlNAM2IR vector was mobilized into the binary vector pART27 to generate pART27-OP:SlNAM2IR.

### Transformation of tomato plants

The binary vectors pART27-OP:SlNAM2, pART27-OP:mSlNAM2, and pART27-OP:SlNAM2IR were transformed into tomato cv. M82 as described previously by Stav *et al.* (2010). Transgenic progeny were selected by germinating sterile seeds on selective medium (1× MS medium, 3% w/v sucrose, 100mg l^–1^ kanamycin), where only transgenic seedlings developed a branched root system. Further validation was performed by PCR of genomic DNA with the primer pairs OCS_rev and SlNAM2_miR164_target_fwd or SlNAM2_mMiR164_target_fwd to detect the *OP:SlNAM2* and *OP:mSlNAM2* transgenes, respectively and with the primer pair pFlap_intron_fwd and SLNAM2IR_ClaI-PstI_fwd to detect the *OP:SlNAM2IR* transgene.

### Real-time quantitative (q) RT-PCR analyses

Total RNA was extracted from different tomato tissues as described above. Total RNA samples were treated with RNase-free DNase (Fermentas Life Sciences, Vilnius, Lithuania) to eliminate genomic DNA contamination. The concentration and integrity of the RNA samples were determined by an ND1000 spectrophotometer (Nanodrop Technologies, Montchanin, DE, USA) and by gel analysis, respectively. First-strand cDNA was synthesized from 2 µg of total RNA using the Maxima First Strand cDNA Synthesis Kit for RT-qPCR (Thermo Scientific) following the manufacturer’s instructions. An RT-negative control was used to ensure the absence of genomic DNA template in the samples. The PCRs were performed with Platinum SYBR Green qPCR Super Mix-UDG (Invitrogen) in a Rotor-Gene 6000 cycler (Qiagen). To ensure the specificity of the amplified fragment, the amplicons were verified by sequencing. Furthermore, at the end of each PCR run, the melting temperature of the product was determined to verify the specificity of the amplified fragment. PCR products were analysed using Rotor Gene Series 6000 software version 1.7 (Qiagen). Two to three independent biological replicates were used for each sample (as indicated), and quantifications were performed in triplicate. The relative expression levels of *GOB, SlNAC1, SlNAM2*, and *SlNAM3* mRNA were calculated using a two-standard curve method normalized to *TIP41* as a reference gene.

### Histological analysis

Analysed tissues were fixed in FAA (Formalin, Acetic acid, Alcohol) until use, then dehydrated in increasing concentrations of ethanol, cleared with histoclear, and embedded in paraffin. Sections cut by microtome to 10 µm thickness were placed on microscope slides and stained with 1% (w/v) Safranin followed by 0.2% (w/v) Fast Green. Slides were examined under bright-field using a Leica light microscope equipped with a camera.

### *In situ* hybridization

Tissue fixation and *in situ* hybridization were performed as described previously by Hendelman *et al.* (2012). For the *in situ* probe, the PCR-amplified SlNAM2 3’ UTR fragment, which was used as siRNA probe, was used as a template for *in vitro* transcription of an antisense cRNA probe with digoxigenin-11-UTP (Roche, Manneim, Germany) using AmpliScribe T7 High Yield Transcription Kit (Epicentre Biotechnologies) according to the manufacturer’s protocol.

## Results and discussion

### Sly-miR164 guides the cleavage of four NAC-domain genes in tomato

**Fig. 1.**
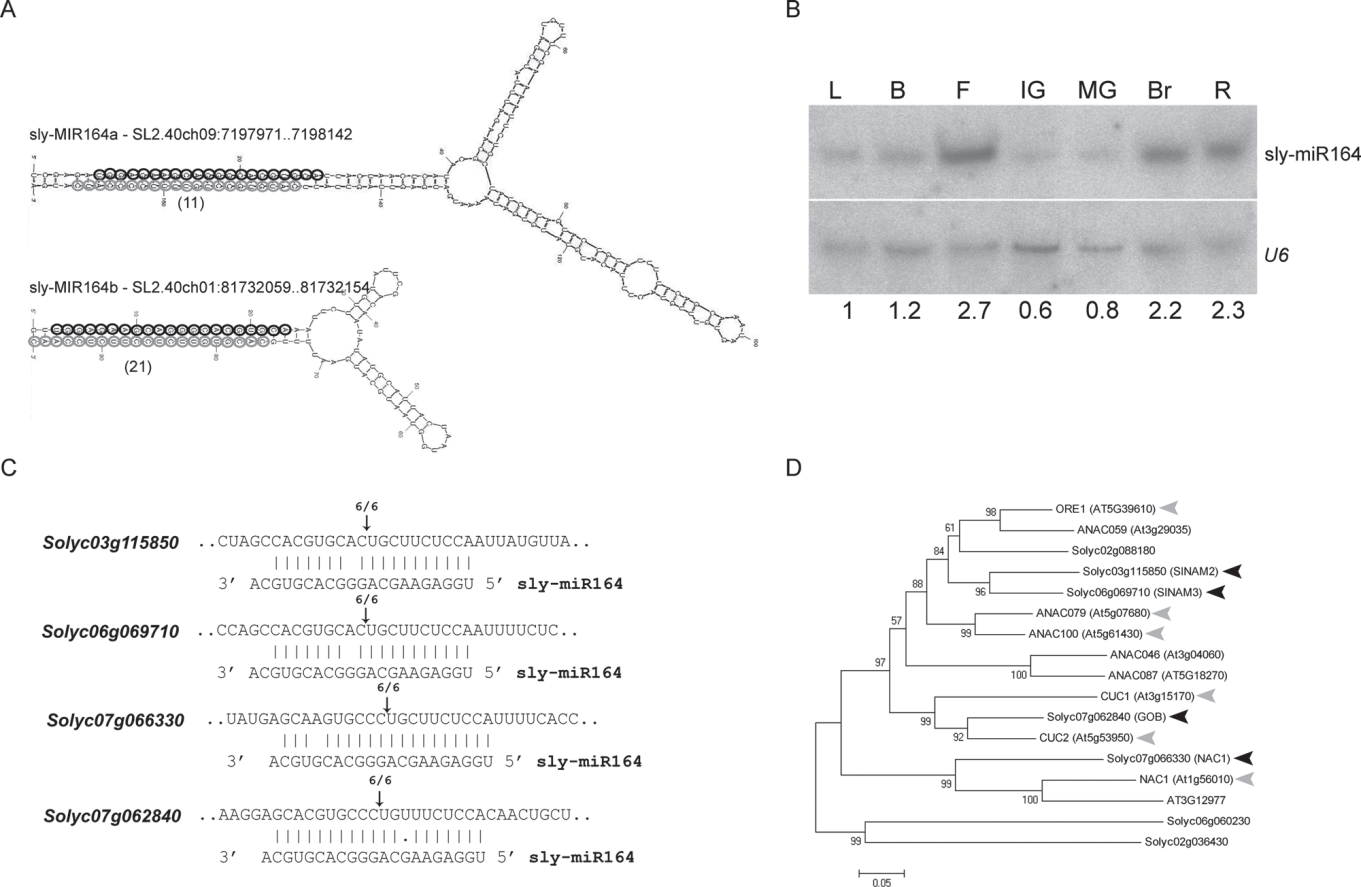
Characterization of sly-miR164 and its target genes in tomato. (A) Hairpin secondary structures of sly-miR164 precursors. The position of each precursor in the tomato genome is indicated. Mature sly-miR164 and matching sly-miR164* sequences are marked by grey and black circles, respectively, and the abundance of sly-miR164* in the seedling small RNA data set is indicated below. (B) Northern blot analysis of sly-miR164 in the indicated tomato tissues. Sly-miR164 expression was normalized to *U6 snRNA* and levels are indicated below the panel. L, leaves; B, buds; F, flower at anthesis; IG, immature green; MG, mature green; Br, breaker; R, red. (C) Experimental validation of sly-miR164 cleavage site in *Solyc03g115850, Solyc06g069710, Solyc07g066330*, and *Solyc07g062840* mRNAs by RLM-RACE. Alignment of sly-miR164 with its target mRNAs. Arrows and numbers indicate the inferred cleavage sites and the fractions of cloned PCR products terminating at this position, respectively. (D) An unrooted phylogenetic tree of NAM family proteins from *Arabidopsis* and tomato, which contain at least one of the motif signatures LPPLxD and [E/x][H/x]VxCFS[N/x]. The phylogenetic tree was constructed by the Neighbor–Joining method with 100 bootstrap sampling (MEGA program, version 4.0) (Tamura *et al.*, 2007). Black and grey arrowheads mark the proteins encoded by sly-miR164 and ath-miR164 -targeted genes, respectively.

MiR164 is a conserved, important regulator of the *CUC* genes which are involved in vegetative as well as reproductive organ-boundary formation (Aida *et al.*, 1997; Laufs *et al.*, 2004). BLASTN with mature miR164 sequences (miRBase, release 19, http://www.mirbase.org/, last accessed 09 September 13) versus our tomato deep-sequenced small RNA data set (Hendelman *et al.*, 2013) and the publicly available tomato small RNA sequences (Tomato Functional Genomics Database) revealed two putative miR164-like sequences (data not shown), but only the two genomic loci encoding the ath-miR164a-identical ones could fold into a pre-miRNA-like hairpin structure (Fig. 1A; see Supplementary Fig. S1A at *JXB* online). In addition, the corresponding sly-miR164* strand encoded by each hairpin was identified in our small RNA data set, validating their functionality as sly-miR164 precursors (Fig. 1A). An additional query of the tomato genome did not identify any novel miR164 sequences suggesting that the identified sly-miR164 is the only miR164 family member encoded by the tomato genome. RNA gel blot was used to analyse the expression of sly-miR164 in vegetative and reproductive tomato tissues. This analysis indicated that sly-miR164 is most abundant in open flowers and ripen fruit (Fig. 1B). In tomato, sly-miR164 has been found to negatively regulate the *CUC2*-like transcription factor *GOB* (Berger *et al.*, 2009). To identify additional NAC-domain genes that are subjected to sly-miR164-guided cleavage in flowers, candidate mRNA targets were predicted and their cleavage was validated by RLM-RACE. This analysis confirmed that in addition to *GOBLET* (*Solyc07g062840*), three mRNA targets—*Solyc03g115850, Solyc06g069710*, and *Solyc07g066330*—were guided to cleavage by sly-miR164 in tomato flowers (Fig. 1C). Sequence analysis of their putative open reading frames indicated that they encode NAC-domain proteins. In addition, this analysis revealed that they all contain the signature motifs LPPLxD and [E/x][H/x]VxCFS[N/x] in their C-terminal region, which predict the involvement of NAC-domain proteins in developmental programmes (see Supplementary Fig. S1B at *JXB* online) (Ooka *et al.*, 2003). Phylogenetic reconstruction of the corresponding tomato and *Arabidopsis* NAC-domain proteins indicated that Solyc07g066330 (SlNAC1) encodes a homologue of *Arabidopsis* miR164-regulated NAC1 which has been found to mediate auxin signalling and to promote lateral root development (Xie *et al.*, 2000; Guo *et al.*, 2005); the related Solyc03g115850 (SlNAM2) and Solyc06g069710 (SlNAM3) proteins (60%/70% identity/similarity), which belong to the same group as the CUC proteins, were distantly related to ORESARA1 (ORE1) which has been found to positively regulate ageing-induced cell death in *Arabidopsis* leaves (Fig. 1D) (Kim *et al.*, 2009).

### Flower-specific silencing of sly-miR164 target genes disturbs whorl and sepal separation

**Fig. 2.**
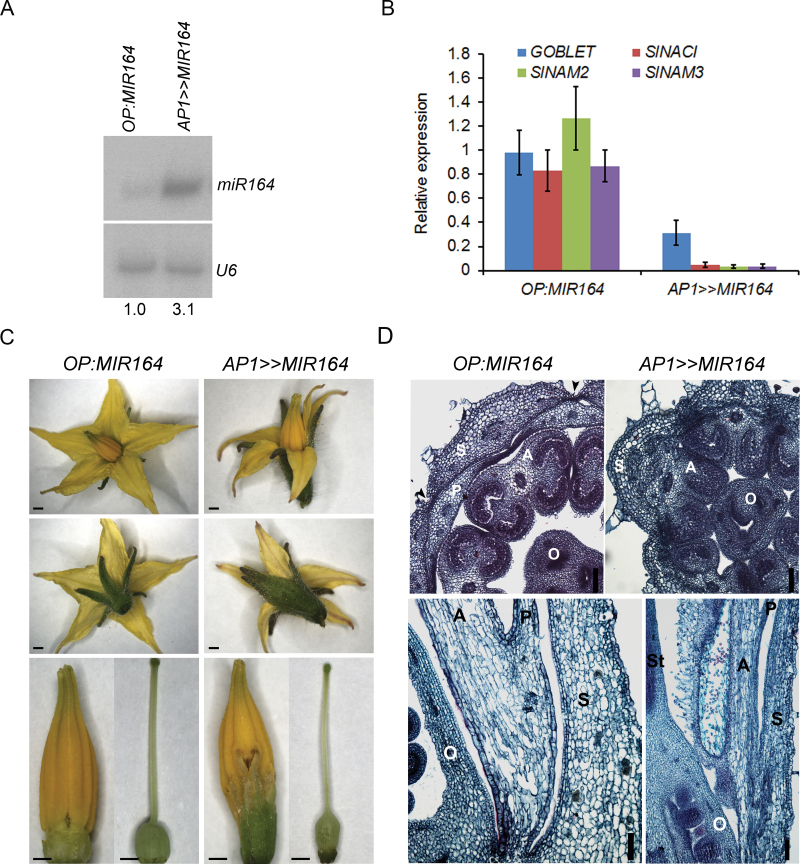
Flower-specific miR164 over-expression leads to sepal and whorl fusions. (A) Northern blot analysis of sly-miR164 in stage 8 buds from the indicated genotypes (tomato flower stages are according to Brukhin *et al.*, 2003). MiR164 expression was normalized to *U6 snRNA* and levels are indicated below the panel. (B) Quantitative RT-PCR analysis of *GOBLET, SlNAC1, SlNAM2*, and *SlNAM3* in stage 8 buds of the indicated genotypes. Primers were designed around the corresponding sly-miR164- complementary site. *TIP41* expression values were used for normalization. Data are means ±SD of three biological replicates, each measured in triplicate. (C) Flower phenotypes of the indicated genotypes. The upper panel presents a whole flower; the middle panel presents a side view of the whole flower, and the lower panel presents isolated anthers and pistil after removal of the sepals and petals. Scale bars=1mm. (D) Transverse and longitudinal sections of control (*OP:MIR164*) and *AP1>>MIR164* stage 10 buds (upper panel) and flowers before anthesis (lower panel). Black arrowheads indicate the sepal–sepal boundary. S, sepal; P, petal; A, anther; O, ovary; St, style. Scale bars=100 µm.

To investigate the involvement of the sly-miR164-targeted *NAM* genes in flower-boundary formation, sly-miR164 was over-expressed in the flower primordia by transactivation of the previously characterized M82 tomato *OP:MIR164* responder line with the available flower-specific *AP1:LhG4* driver line, which drives expression throughout young floral primordia (Hendelman *et al.*, 2013). First, sly-miR164 over-expression was validated by Northern analysis of young *AP1>>MIR164* buds revealing a 3-fold increase in its levels compared with control buds (Fig. 2A). This increase was consistent with the significant reduction in *GOB* (70%) and *SlNAC1*, *SlNAM2*, and *SlNAM3* (~95%) accumulation in these buds, further corroborating their targeting by sly-miR164 (Fig. 2B). Phenotypic analysis of silenced *AP1>>MIR164* flowers revealed elongated sepals that were fused to each other at various points (Fig. 2C). Moreover, failure of these sepals to peel away from the flower suggested the occurrence of partial fusion between the first and second whorls (Fig. 2C). Indeed, transverse sectioning of young *AP1>>MIR164* buds at the base of the style showed that the three outer whorls and their organs were not separated at that stage whereas, in control buds, they were completely separated (Fig. 2D). Accordingly, longitudinal sectioning of fully developed *AP1>>MIR164* flowers showed that the three outer whorls and, in addition, the fourth whorl separated later than in controls (Fig. 2D). Together, these phenotypes indicated that sly-miR164 target genes are required for the normal formation of flower sepal and interwhorl boundaries. The *gob-3* loss-of-function tomato mutant has been shown to produce flowers with increased sepal fusions and fewer locules, and to set fruit with fused outer floral organs, suggesting that *GOB* is central to the formation of tomato-flower boundaries (Blein *et al.*, 2008; Berger *et al.*, 2009). Thus, it is highly likely that the reduced levels of *GOB* in *AP1>>MIR164* flower primordia are responsible for at least some of the defective boundary phenotypes. Nevertheless, since *GOB* silencing in *AP1>>MIR164* flowers was driven by a heterologous promoter and was not complete as in the *gob-3* loss-of-function mutant flowers, the *AP1>>MIR164* defective flower-boundary phenotypes might be the result of the down-regulation of *GOB* and either one or a combination of the other sly-miR164 target genes.

### SlNAM2 is expressed in floral boundaries

**Fig. 3.**
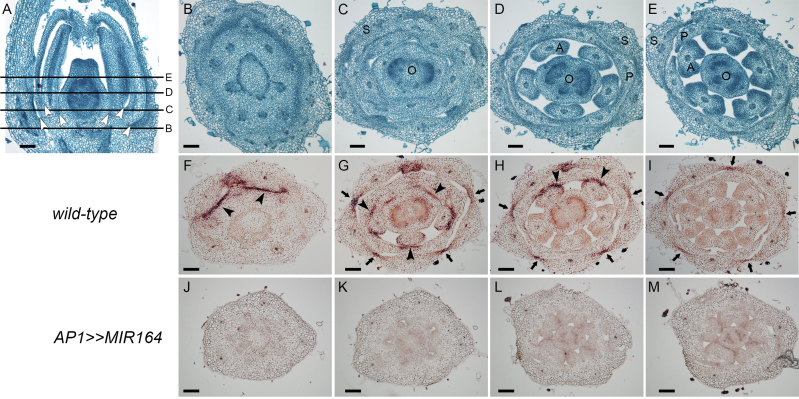
Expression pattern of *SlNAM2* in tomato bud. (A) Longitudinal section of wild-type flower buds at stage 8. The lines mark the positions of the cross-sections shown in (B)–(E). Arrowheads mark the points of separation between corresponding whorls. (B–M) Successive transverse sections from the base upward. (B–E) Safranin-Fast Green differential staining. (F–M) *In situ* hybridization with *SlNAM2* antisense probe in the indicated genotypes. Arrowheads and arrows mark *SlNAM2* signal between whorls and around floral organs, respectively. S. sepal; P, petal; A, anther; O, ovary. Scale bars=100 µm.

Since *SlNAC1* probably represents a homologue of *Arabidopsis NAC1* (Fig. 1D), which has not been implicated in flower-boundary formation (Guo *et al.*, 2005), its contribution to the boundary-defective phenotype was less likely. In addition, *SlNAM2* was much more abundant than *SlNAC1* and *SlNAM3* in developing flowers (see Supplementary Fig. S2 at *JXB* online). Thus, to examine the possible involvement of *SlNAM2* in flower-boundary establishment, its spatial expression in young buds was determined by *in situ* hybridization. Wild-type M82 tomato flowers are composed of four whorls of distinct floral organs. Longitudinal and successive transverse sections of stage 8 buds showed the spatial separation of the whorls and their corresponding floral organs (Fig. 3A–E). A transverse section of a relatively proximal part of a young bud showed completely fused whorls (Fig. 3B). At that same location, stripes of *SlNAM2* mRNA were expressed at the boundary between the first and second whorls prior to their separation (Fig. 3F). In a more distal plane, the first and the fourth whorl are clearly separated from the second and third, respectively, but the perianth organ primordia are still laterally fused (Fig. 3C). At that position, stripes of *SlNAM2* mRNA were detected at the boundaries between the second and the third and the third and the fourth whorls. In addition, *SlNAM2* mRNA marked the lateral margins of the sepals and surrounded the stamen filaments (Fig. 3G). At a more distal plane, all whorls were separated (Fig. 3D) and *SlNAM2* mRNA was detected between the fused sepals (Fig. 3H). In the most distal section of the ovary, the sepals, which protect the bud, were the only organs that remained fused to each other (Fig. 3E) and that fusion was marked by *SlNAM2* mRNA (Fig. 3I). In accordance with its strong sly-miR164-mediated silencing in *AP1>>MIR164* buds (Fig. 2B), *SlNAM2* expression was not detected in them, further confirming the authenticity of the wild-type *in situ* signal (Fig. 3J–M). Taken together, the *SlNAM2* transcript was expressed at the boundaries between adjacent sepals and whorls suggesting that it might be involved in their separation. However, no significant *SlNAM2* mRNA signal could be detected before the fusion of carpels (bud stages 1–6; data not shown), indicating that it is poorly expressed at the time of whorl-boundary formation. *GOB* is expressed in the boundaries between the floral meristem and floral-organ primordia (Blein *et al.*, 2008). This implicates *GOB* rather than *SlNAM2* in whorl boundary formation and raises the possibility that *SlNAM2* is involved in floral-boundary maintenance.

### Accumulation of *SlNAM2*-encoding transcript is associated with growth-repression phenotypes

Cell proliferation is greatly reduced in the organ–meristem and organ–organ boundaries (Breuil-Broyer *et al.*, 2004). This process is mediated by the activity of regulatory boundary genes (Aida and Tasaka, 2006*a*) and plays a role in organ morphogenesis (Nikovics *et al.*, 2006). While loss-of-function mutations in these genes result in overgrowth of the boundary region, manifested as organ fusions, over-accumulation of these genes due to gain-of-function or ectopic expression usually represses growth, manifested as smaller and occasionally multiple organs and extra and elaborate lobing of cotyledons, leaves, and floral organs (Hiratsu *et al.*, 2002; Brewer *et al.*, 2004; Baker *et al.*, 2005; Berger *et al.*, 2009; Busch *et al.*, 2011; Huang *et al.*, 2012).

**Fig. 4.**
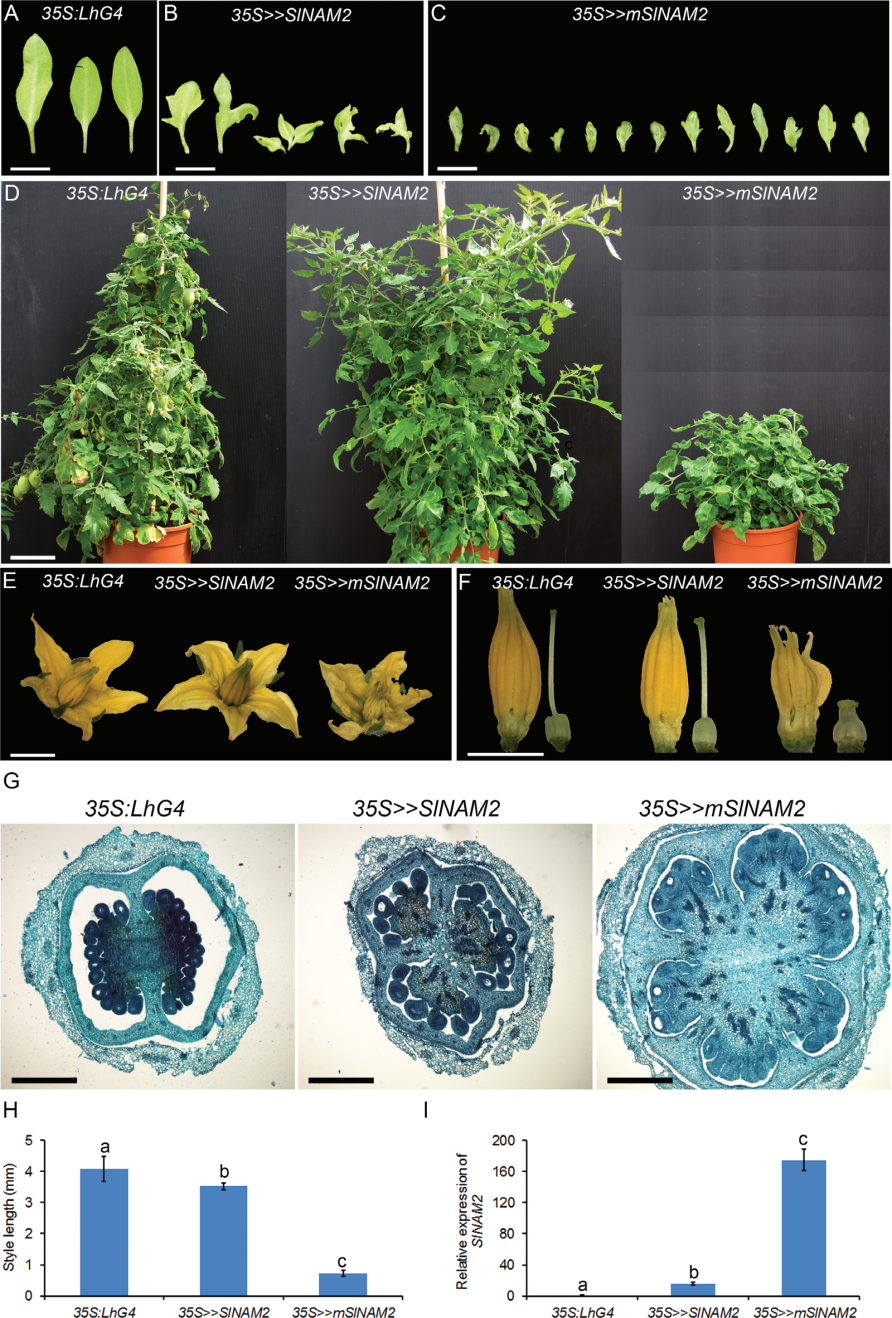
Phenotypic and molecular characterization of *35S>>SlNAM2* and *35S>>mSlNAM2* plants. (A–C) Phenotypes of isolated cotyledons 11 d after sowing from control (*35S:LhG4*) and transactivated (*35S>>SlNAM2* and *35S>>mSlNAM2*) tomato seedlings. Multiple cotyledons were separated. (D) Four-month-old plants of the indicated genotypes. (E) Representative flower at anthesis of the indicated genotypes. (F) Stamen and pistil phenotypes of the indicated genotypes. (G) Transverse sections of the ovary of the indicated genotypes. (H) Style lengths of the indicated genotypes. Data are means ±SD (*n*=10). Different letters indicate statistically significant difference as determined by Student’s *t* test (*P* ≤0.01). (I) QRT-PCR analysis of SlNAM2-encoding transcripts in the flowers of indicated genotypes. Primers were designed around the corresponding sly-miR164 complementary site. *TIP41* expression values were used for normalization. Data are means ±SD of two biological replicates, each measured in triplicate. Different letters indicate statistically significant difference as determined by Student’s *t* test (*P* ≤0.01). Scale bars: (A–C)=1cm; (D)=10cm; (E, F)=5mm; (G)=500 µm.

Since *SlNAM2* is expressed at flower boundaries, the question was asked whether it has similar boundary gene activity and can suppress growth when accumulated. To investigate this, two homozygous tomato responder lines were generated that are able to express wild-type (*SlNAM2*) and sly-miR164-resistant (*mSlNAM2*) versions of the gene upon transactivation (for further details see the Material and methods and see Supplementary Fig. S3 at *JXB* online). Both were crossed with the strong constitutive *35S:LhG4* and flower-specific *AP1:LhG4* driver lines to generate corresponding transactivated F_1_ progeny plants. Inactivated *OP:SlNAM2* and *OP:mSlNAM2* responder plants were morphologically identical to the driver lines and wild-type M82 tomato (data not shown). However, *35S>>mSlNAM2* plants and, to a much lesser extent, *35S>>SlNAM2* plants showed various growth-repression-associated phenotypes. Compared with control tomato cotyledons which are oval and entire, the *35S>>mSlNAM2* cotyledons were abnormally shaped, smaller and lobed, and occasionally three instead of two cotyledons were produced (Fig. 4A–C). A similar but less pronounced phenotype was observed in *35S>>SlNAM2* cotyledons, which were larger than *35S>>mSlNAM2* (Fig. 4B). Reminiscent multiple and serrated cotyledon phenotypes have also been reported as a result of expression of the *GOB* sly-miR164-resistant mutant gene *Gob-4d* under its native or leaf-specific *FIL* promoter, respectively (Berger *et al.*, 2009). In addition, mature *35S>>mSlNAM2* plants were dwarf whereas the *35S>>SlNAM2* plants were no different from the controls (Fig. 4D). Moreover, examination of *35S>>mSlNAM2* flowers revealed a reduction in flower size and wrinkled and slightly lobed petals (Fig. 4E). Also, compared with the control and *35S>>SlNAM2*, dramatic growth repression was observed in the two inner whorls of *35S>>mSlNAM2* flowers, including shorter stamens and style (Fig. 4F, H). Moreover, the pistil was very wide as a result of extra carpel formation (Fig. 4G). QRT-PCR analysis of control and transgenic flowers revealed the relatively mild accumulation of *SlNAM2* in *35S>>SlNAM2* compared with the controls and, consistent with *mSlNAM2* resistance to sly-miR164 cleavage, a much higher accumulation of SlNAM2-encoding transcript was detected in *35S>>mSlNAM2* flowers (Fig. 4I).

**Fig. 5.**
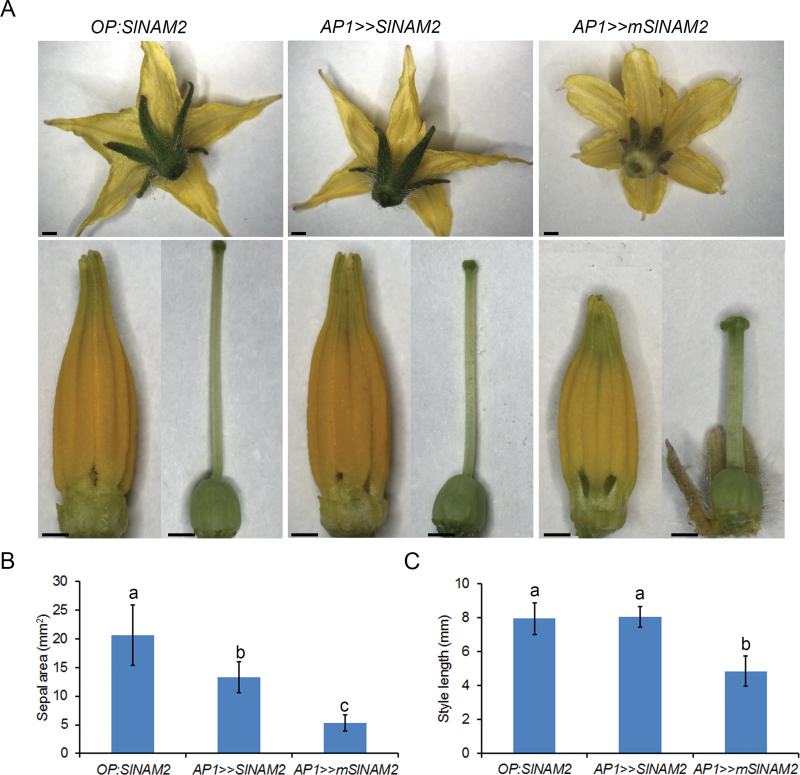
Phenotypic characterization of *AP1>>SlNAM2* and *AP1>>mSlNAM2* flowers. (A) Representative flower at anthesis of the indicated genotypes. The upper panel presents the whole flower and the lower panel presents the anthers and pistil after the sepals and petals were removed. Scale bars=1mm. (B) Sepal areas of the indicated genotypes. Data are means ±SD (*n* ≥70). Different letters indicate statistically significant difference as determined by Student’s *t* test (*P* ≤0.01). (C) Style lengths of the indicated genotypes. Data are means SD (*n* ≥10). Letters indicate statistically significant differences as determined by Student’s *t* test (*P* 0.01).

Similarly, high accumulation of SlNAM2-encoding transcript in *AP1>>mSlNAM2* buds (see Supplementary Fig. S4 at *JXB* online) was associated with significantly smaller sepals and styles and slightly lobed petals compared with the organs of control *OP:SlNAM2* flowers (Fig. 5), whereas less accumulation in *AP1>>SlNAM2* buds resulted in milder organ phenotypes (Fig. 5). Together, these results demonstrated a positive correlation between the accumulation levels of SlNAM2-encoding transcript and abnormalities typically observed in plants over-expressing boundary genes, suggesting similar activity for *SlNAM2*.

### *SlNAM2* accumulation rescues the fusion phenotypes of AP1>>MIR164 flowers

**Fig. 6.**
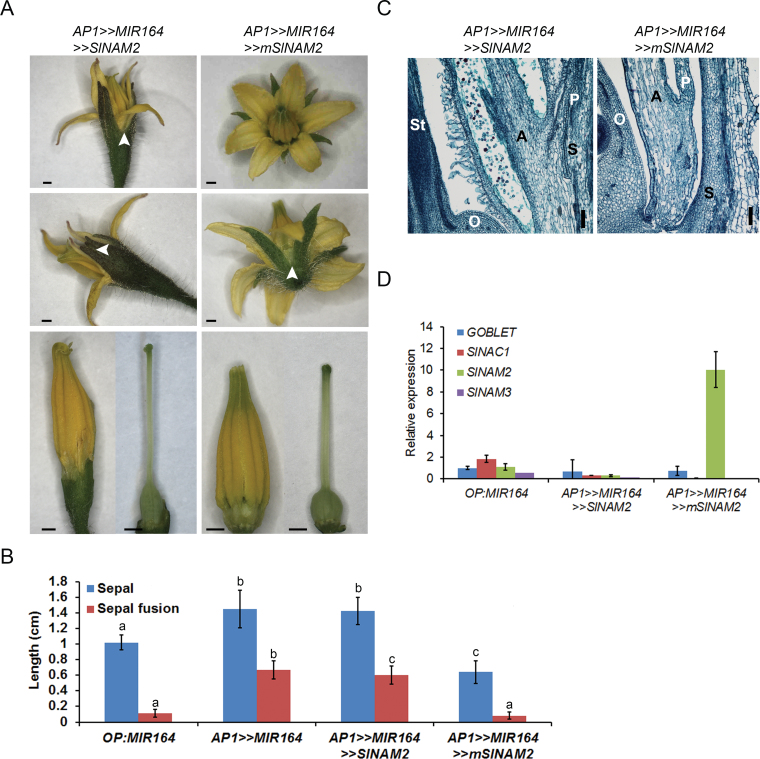
Restoration of normal flower phenotype upon expression of *mSlNAM2* in miR164-over-expressing flowers. (A) Flower phenotypes of the indicated genotypes. The upper panel presents the whole flower; the middle panel presents a whole flower on its side, and the lower panel presents isolated anthers and pistil. Arrowheads mark the points of sepal fusion. Scale bars=1mm. (B) Sepal and fusion lengths of the indicated genotypes. (C) Longitudinal section of flowers before anthesis from the indicated genotypes. Scale bars=100 µm. S, sepal; P, petal; A, anther; O, ovary; St, style. (D) Quantitative RT-PCR analysis of *GOBLET, SlNAC1, SlNAM2,* and *SlNAM3* in 1–2mm buds of the indicated genotypes. Primers were designed around the corresponding miRNA’s complementary site. *TIP41* expression values were used for normalization. Data are means ±SD of three biological replicates, each measured in triplicate.

Our results indicated that *SlNAM2* is expressed at floral whorl and organ boundaries and might suppress growth when accumulated. In the absence of an informative loss-of-function mutant (see Supplementary Fig. S5 at *JXB* online), the question was then asked whether SlNAM2 growth suppression activity can define floral boundaries. To that end, *AP1>>MIR164* mutant flowers, which had fused sepals and abnormal whorl separation, were complemented with SlNAM2 and the resulting phenotype was analysed. This was done by expressing *mSlNAM*2 in the background of *AP1>>MIR164* plants (*AP1>>MIR164 >>mSlNAM2*). As a control, *SlNAM2* was expressed on the same genetic background (*AP1>>MIR164 >>SlNAM2*). As expected, analysis of *AP1>>MIR164 >>SlNAM2* flowers showed elongated fused sepals and abnormal interwhorl fusion (Fig. 6A–C). This abnormal phenotype was no different from that of *AP1>>MIR164* flowers (Fig. 2C, D). By contrast, the *AP1>>MIR164 >>mSlNAM2* flowers had a wild-type-like phenotype. Although their sepals were slightly shorter than controls they were not fused (Fig. 6A, B) and contained no abnormal interwhorls fusions (Fig. 6A, C). QRT-PCR of young buds revealed accumulation of the sly-miR164-ressistant *mSlNAM2* in *AP1>>MIR164 >>mSlNAM2* whereas the sly-miR164-sensitive *SlNAM2*, *SlNAC1*, *SlNAM3*, and *GOB* were silenced (Fig. 6D). These results demonstrated that, when precociously expressed, SlNAM2 is able to restore the formation of floral boundaries.

In *Arabidopsis* leaves, complementation of the *cuc2* mutant by a NAC-domain gene did not occur unless they had redundant functions. *NAC1*, *ANAC019*, and to some extent, *CUC3*, which have different functionalities from that of *CUC2*, were not able to restore leaf morphogenesis whereas the redundant gene, *CUC1*, could (Hasson *et al.*, 2011). Thus, the boundary restoration ability of SlNAM2 might reflect some functional redundancy with *GOB*, which was suggested to be required for the formation of tomato flower boundaries (Blein *et al.*, 2008). Still, the relative over-accumulation of SlNAM2-encoding transcripts needed for boundary restoration and the milder phenotypes of *35S>>mSlNAM2* compared with *35S>>Gob-4d* plants (Berger *et al.*, 2009), suggest that *SlNAM2* activities are less suitable for boundary formation than *GOB*.

*Arabidopsis* leaf serration occurs in two different phases: an early phase, requiring *CUC2*, during which the boundaries which surround the emerging tooth are initiated, and a later phase, requiring both *CUC2* and *CUC3*, which maintains the boundaries to sustain teeth formation (Hasson *et al.*, 2011). In accordance with that, *CUC2* is already expressed in the leaf primordium margins before teeth outgrowth whereas *CUC3* can hardly be detected at that stage and, afterwards, both are detected in the sinuses of the developing serrations (Nikovics *et al.*, 2006; Hasson *et al.*, 2011). In a reminiscent way, the non-overlapping expression patterns of *GOB* (Blein *et al.*, 2008) and *SlNAM2* in floral boundaries (Fig. 3), where *GOB* precedes *SlNAM2* expression, may suggest that they function at different stages of boundary morphogenesis. The occurrence of *SlNAM2* in the floral-boundary after they were initiated by *GOB* might imply its function in boundary maintenance rather than in their formation. However, to support this hypothesis, the analysis of the *SlNAM2* loss-of-function mutant is required.

## Supplementary data

Supplementary data can be found at *JXB* online.

Supplementary Table S1. Primers used in this study.

Supplementary Fig. S1. Sequence alignment of sly-miR164-targeted NAC transcription factors.

Supplementary Fig. S2. Quantitative RT-PCR analysis of sly-miR164-targeted genes in developing flowers.

Supplementary Fig. S3. Generation of *OP:SlNAM2* and *OP:mSLNAM2* responder lines.

Supplementary Fig. S4. Quantitative RT-PCR analysis of *SlNAM2* in stage 9 buds.

Supplementary Fig. S5. Molecular analysis of *35S>>SlNAM2IR* plants.

Supplementary Data
