# Supplementary material for: The tomato NAC transcription factor SlNAM2 is involved in flower-boundary morphogenesis
Source: J Exp Bot. 2013 Oct 1;64(18):5497–507. doi: 10.1093/jxb/ert324 (PMC3871814; doi:10.1093/jxb/ert324)
Supplement: Supplementary Data [file supp_64_18_5497__index.html]

The tomato NAC transcription factor SlNAM2 is involved in flower-boundary morphogenesis — The tomato NAC transcription factor SlNAM2 is involved in flower-boundary morphogenesis — The tomato NAC transcription factor SlNAM2 is involved in flower-boundary morphogenesis — Supplementary Data 

# The tomato NAC transcription factor *SlNAM2* is involved in flower-boundary morphogenesis

## Supplementary Data

Data files

**Files in this Data Supplement:**

- Supplementary Data - Supplementary Data
