# Supplementary material for: The tomato NAC transcription factor SlNAM2 is involved in flower-boundary morphogenesis
Source: J Exp Bot. 2013 Oct 1;64(18):5497–507. doi: 10.1093/jxb/ert324 (PMC3871814; doi:10.1093/jxb/ert324)
Supplement: Supplementary Data [file supp_ert324_jexbot107086_file001.pdf]

Supplementary Table S1. Primers used in this study

**Primer ID**	**Primer sequence (5' – 3')**[abc]
GeneRacer-5'	CGACTGGAGCACGAGGACACTGA
GeneRacer-5'-nested	GGACACTGACATGGACTGAAGGAGTA
SlNAM2_RACE	GCAAATTCCTTAGCGTTGCGGGATCTTGTAT
SlNAM3_RACE	TCAGCAACTCCAGAGACAATCAAGACCCTGTA
SlNAC1_RACE	TCACACGTAGAGGATGTTGCAGTGGCATT
GOBLET_RACE	TTTCTGAGTCTCCGGCACGGTCCAATTA
SlNAM2_RACE_nested	TGGTTCCAGGTGAACGAAGTCGGAAGA
SlNAM3_RACE_nested	TGAAGGATCTTGAACCCCAAATGAAGCTGGA
SlNAC1_RACE_nested	GTATGCGGTAGATCCGATGGCGGTTG
GOBLET_RACE_nested	CCGCCGGAGAATAACGGAAGCTGGA
OCS_rev	GAAACCGGCGGTAAGGATCT
SlNAM2_Exon_557_fwd	TCCTAAAACAGTAAAGAATGATTGG
XhoI-U218896_fwd	CCGCTCGAGGTTGTTTTTG*ATG*ATGATGGA
BamHI-U218896_rev	CGGGATCC*TCA*GTAAGTCCAGAAGCAATCAAGA
164-mutant-target_fwd	TAGCCACGTGCAtTGtTTtagtAATTATGTTACTACTCAAAAGAAT
164-mutant-target_rev	AATTactaAAaCAaTGCACGTGGCTAGGCGATGAAT
SlNAM2_miR164_target_fwd	CTGCTTCTCCAATTATGTTACTACTC
SlNAM2_mMiR164_target_fwd	TTGTTTTAGTAATTATGTTACTACTCAAAAGA
UBI3_rev	TCCCAAGGGTTGTCACATACATC
UBI3_fwd	AGAAGAAGACCTACACCAAGCC
SlNAM2IR_ClaI-PstI_fwd	CCATCGATTGCACTGCAGTGCAGATCTTGATTGCTTCTGGACTTACTG
SlNAM2IR_HindIII-EcoRI_rev	CCCAAGCTTGGGGGAATTCCGTAAGCCAACCAACCTGATCTC
U6 probe	AGGGGCCATGCTAATCTTCTC
pGEM-T7	TAATACGACTCACTATAGGG
pFlap_intron_fwd	AATTTCTTGTTTCCGATCCTCATA
qRT-SlNAM2_fwd	CCACCATTGACAGATTCATCG
qRT-SlNAM2_rev	GGTGAACGAAGTCGGAAGAG
qRT-GOBLET_fwd	TCGATTCCTCTCCGTATAGCAC
qRT-GOBLET_rev	GTCGAAGACAGAAGTTGGATCG
qRT-SlNAC1_fwd	CGACCAAACAAACCCTAACAAC
qRT-SlNAC1_rev	TGGTTAGGGGTGAAAATGGAG
qRT-SlNAM3_fwd	ACTGCTACTGCTTCGAAATCCA
qRT-SlNAM3_rev	TGAATGGAGCTATTTGGTTACAAGA
qRT-TIP41_fwd	ATGGAGTTTTTGAGTCTTCTGC
qRT-TIP41_rev	GCTGCGTTTCTGGCTTAGG

[a]Added restriction enzyme sites are underlined.

[b]lowercase letters indicate substituted nucleotides

[c]Italics letters indicate stop or start codon.

Journal of Experimental Botany, The tomato NAC transcription factor *SlNAM2* is involved in flower-boundary morphogenesis

Anat Hendelman, Ran Stav, Hanita Zemach and Tzahi Arazi

Fig. S1. Sequence alignments of sly-miR164 and sly-miR164-targeted NAC transcription factors. (A) Nucleotide sequence alignment of *Arabidopsis* (ath), rice (osa), *Medicago truncatula* (mtr), *Sorghum bicolor* (sbi) and tomato (sly) miR164 members. Identical nucleotides are shaded in black. (B) The multiple alignment was generated with the computer program CLUSTALW (Thompson *et al.*, 1994). The conserved NAC domain is marked by asterisks. The sequences that match the LPPLxD and [E/x][H/x]VxCFS[N/x] motif signatures are boxed.

Fig. S2. Quantitative RT-PCR analysis of sly-miR164-targeted genes in developing flowers. Primers were designed around the corresponding miR164 complementary sites. *TIP41* expression values were used for normalization. Data are means ± SD of three biological replicates, each measured in triplicate. Different letters indicate statistically significant differences as determined by Student's *t* test ($P \leq 0.05$).

Fig. S3. Generation of *OP:SlNAM2* and *OP:mSLNAM2* responder lines. (A) Schematic representation of the responder binary constructs. The sly-miR164 complementary sequence in *SlNAM2* mRNA, the sly-miR64 sequence, the silent mutations (in red) introduced in *mSlNAM2* and the minimum free energy hybridization values as determined by RNAhybrid (Rehmsmeier *et al.*, 2004) are shown in the expanded region. (B) Genomic DNA PCR analysis of representative tomato *OP:SlNAM2* and *OP:mSlNAM2* reporter lines. The *OP:SlNAM2#4* and *OP:mSlNAM2#7* lines were used for further analysis. (C) Cleavage analysis of *SlNAM2* and *mSlNAM2* transgenic transcripts. The miRNA-mediated cleavage site was determined by RLM-RACE of total *35S>>SlNAM2* and *35S>>mSlNAM2* leaf RNA using a transgene-specific RACE primer for the 3' OCS. The presence of an intact transgenic transcript was verified by RT-PCR. Below, arrow marks the position of the inferred cleavage site in the transgenic

SlNAM2 transcript, and the number above it indicates the fraction of cloned PCR products terminating at this position.

Fig. S4. Quantitative RT-PCR analysis of *SlNAM2* in stage 9 buds. Primers were designed around the corresponding miR164 complementary site. *TIP41* expression values were used for normalization. Data are means ± SD of two biological replicates, each measured in triplicate. Different letters indicate statistically significant difference as determined by Student's *t* test ($P \leq 0.01$).

Fig. S5. Molecular analysis of *35S>>SlNAM2IR* plants. (A) RNA gel-blot analysis of *SlNAM2* siRNAs in the indicated *35S>>SlNAM2IR* plants (upper panel). The *U6 snRNA* was used as a loading control (lower). (B) Quantitative RT-PCR analysis of *SlNAM2* in the flowers of the indicated *35S>>SlNAM2IR* plants. Primers were designed around the corresponding sly-miR164 complementary site. *TIP41* expression values were used for normalization. Data are means ± SD of three technical replicates. This analysis showed that despite the production of *SlNAM2* siRNAs, the transcript level of *SlNAM2* remained unchanged in their flowers, rendering these plants non-informative.

Rehmsmeier M, Steffen P, Hochsmann M, Giegerich R. 2004. Fast and effective prediction of microRNA/target duplexes. *Rna-a Publication of the Rna Society* **10**, 1507-1517.

Thompson JD, Higgins DG, Gibson TJ. 1994. Clustal-W - Improving the Sensitivity of Progressive Multiple Sequence Alignment through Sequence Weighting, Position-Specific Gap Penalties and Weight Matrix Choice. *Nucleic Acids Research* **22**, 4673-4680.

(A)

```
ath-miR164a   UGGAGAAGCAGGGCACGUGCA
ath-miR164c   UGGAGAAGCAGGGCACGUGCG
mtr-miR164a   UGGAGAAGCAGGGCACGUGCA
osa-miR164a   UGGAGAAGCAGGGCACGUGCA
sbi-miR164a   UGGAGAAGCAGGGCACGUGCA
sly-miR164a   UGGAGAAGCAGGGCACGUGCA
```

(B)

```
                                               *******************************************************************
SlNAM2    MENFSASVKMDDQQQMELPPGFRFHPTDEELITHYLSKKVVDMN---FSAIAIGDVDMNKIEPWELPWKAKIGEKEWYFFCVRDKKYPTGLRTNRATAAG 97
SlNAM3    MENYSGVVKDDDQ--MELPPGFRFHPTDEELITHYLSNKVVDTN---FVAIAIGDVDLNKVEPWDLPWKAKMGEKEWYFFCVRDKKYPTGLRTNRATAAG 95
SlNAC1    MSNNNSLSMVESK----LPPGFRFHPRDEELICDYLMKKVDQQSEYQQQYPLLIEVDLNKSEPWEIPEVACVGGKEWYFYSQRDRKYATGLRTNRATVSG 96
GOBLET    MEIYHQMQFDCGDP--HLPPGFRFHPTDEELITYYLLKKVLDCN---FTARAIAEVDLNKCEPWELPGKAKMGEKEWYFFSLRDRKYPTGLRTNRATEAG 95

          *************************************************************************
SlNAM2    YWKATGKDKEIFRGR--SLVGMKKTLVFYRGRAPRGEKTNWVTHEYRLEGRLSLNNLPKTVKNDWVICRVFQKTTG----------GKKIHISGLVRANS 185
SlNAM3    YWKATGKDREIFRGK--SLVGMKKTLVFYKGRAPKGEKTNWVIHEFRLEGKLSLQNLPKTAKNEWVICRVFQKSSG----------GKKIHISGLLKLNS 183
SlNAC1    YWKATGKDRAIIRKG--SLVGMRKTLVFYQGRAPKGRKSDWVMHEFRLE--LPIRPQISSIKEDWVLCRVFHKKKE---------LLATKQEIGSNNIYY 183
GOBLET    YWKATGKDREIFSSKTCALVGMKKTLVFYRGRAPKGEKSNWVMHEYRLDGKFAYHYISRSSKDEWVISRVFQKSTGSNGAATSTGGGKKRLSSSINMYQE 195

SlNAM2    DENEMVNTVLPPLTDSS------PSHVHCFSNYVTTQKNQENNMINSFNNSPNFPLLSNSIDIFQRNS-LPTSFTWNQNVPLQHNFPQPGSFPIQDP-AT 277
SlNAM3    NENEMGNSFLPPLTDSATATASKSSHVHCFSNFLTAQNN-------------CFPLLSNPMDSYPTTSLVPNTFSCNQIAPFTTTN-NPASFGVQDPSIL 269
SlNAC1    DNDTISCSSLPPLMDPY----------ITFDQTNPNNNNMN-----------MNELYYEQVPCFSIFTPNQTFTSHSHHLPSATTAIGSTAYGGFPADIG 262
GOBLET    VSSPSSVSHLPPLLDSS----PYSTTATSAAAIVIGDRDR------------DHSFKKEHVPCFSTTATATITAQSLTFDPTSVFDISSNTLHALQPTPS 279

SlNAM2    LRNLLEN--YGHQSFKKETDMISVSQETG-ISTDRNTE-----ITSAQ---------------------QDLDCFWTY- 326
SlNAM3    LRTSLDS--YGLN-FKKE-DIFNVPQETGVISTDMNTD-----ITSVVSNLEMKRRFLEDQVPSAGMVGLQGLDCLWSC- 339
SlNAC1    NYLNATATSSTCDNNKVIRAVLSHLSTKNNIIMEGNNSNNNNIINPAQNIKGGNSPSFGEGSSETSFLSEVGYPTMWNNY 342
GOBLET    FASILDSSPSNFTNYTRNSTFPSLRSLHENLQLPLFSG-----GTSAMHG-GFSNPMVNWTVPETQKVEQSELDCMWSY- 352
```

Figure S1

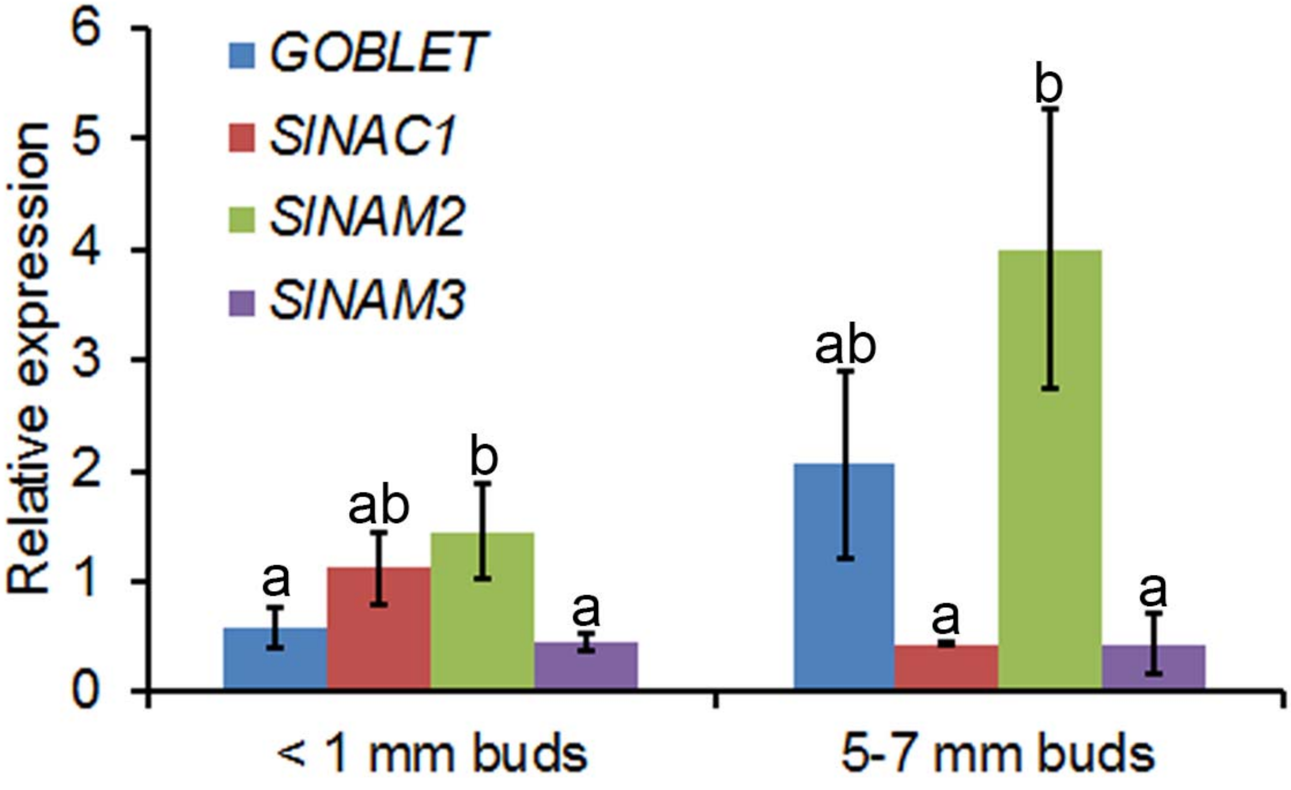

6
5
4
3
2
1
0
Relative expression
GOBLET
SlNAC1
SlNAM2
SlNAM3
a
ab
b
a
ab
a
b
a
< 1 mm buds
5-7 mm buds

Figure S2

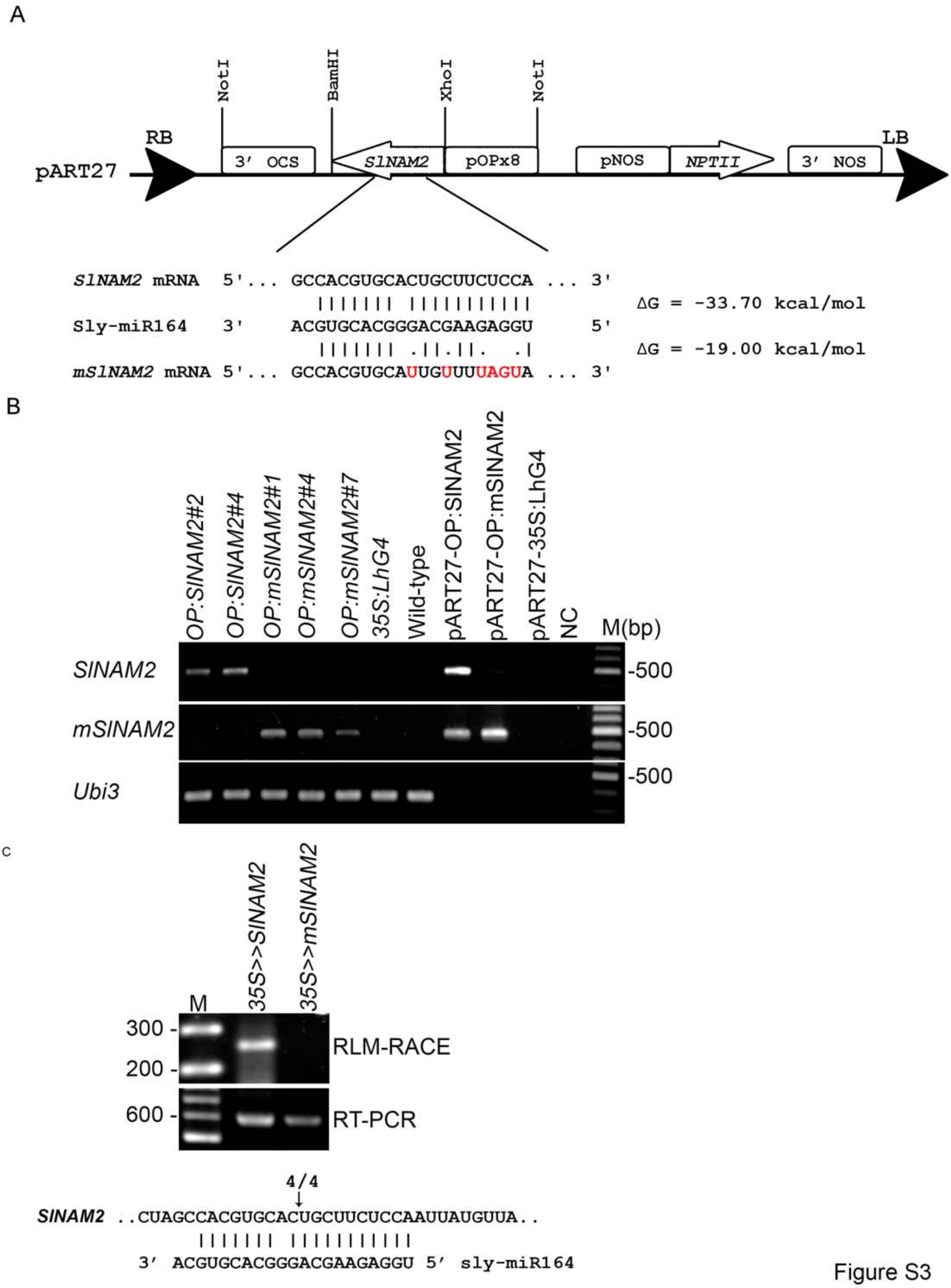
A
NotI
BamHI
XhoI
NotI
RB
LB
pART27
3′ OCS
SlNAM2
pOPx8
pNOS
NPTII
3′ NOS
SlNAM2 mRNA 5'... GCCACGUGCACUGCUUCUCCA ... 3'
ΔG = -33.70 kcal/mol
Sly-miR164 3' ACGUGCACGGGACGAAGAGGU 5'
ΔG = -19.00 kcal/mol
mSlNAM2 mRNA 5'... GCCACGUGCAUUGUUUUAGUA ... 3'
B
OP:SlNAM2#2
OP:SlNAM2#4
OP:mSlNAM2#1
OP:mSlNAM2#4
OP:mSlNAM2#7
35S:LhG4
Wild-type
pART27-OP:SlNAM2
pART27-OP:mSlNAM2
pART27-35S:LhG4
NC
M(bp)
SlNAM2
mSlNAM2
Ubi3
-500
-500
-500
C
M
35S>>SlNAM2
35S>>mSlNAM2
300 -
200 -
600 -
RLM-RACE
RT-PCR
4/4
SlNAM2 ..CUAGCCACGUGCACUGCUUCUCCAAUUAUGUUA..
3′ ACGUGCACGGGACGAAGAGGU 5′ sly-miR164

Figure S3

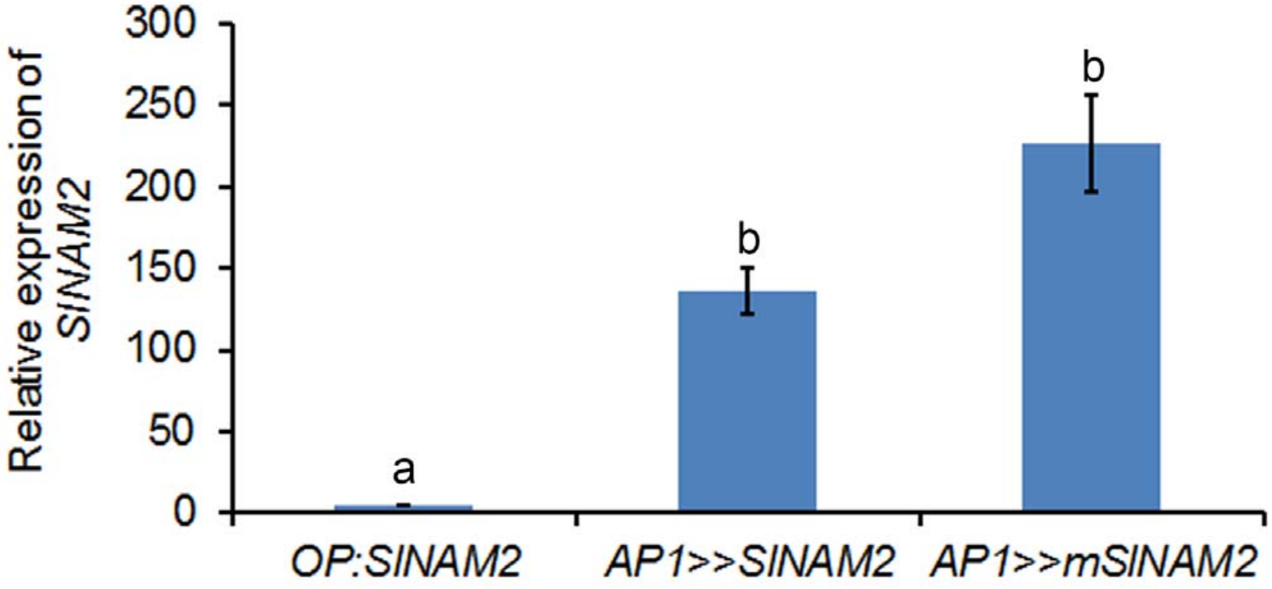
300
250
200
150
100
50
0
Relative expression of SlNAM2
a
b
b
OP:SlNAM2
AP1>>SlNAM2
AP1>>mSlNAM2

Figure S4

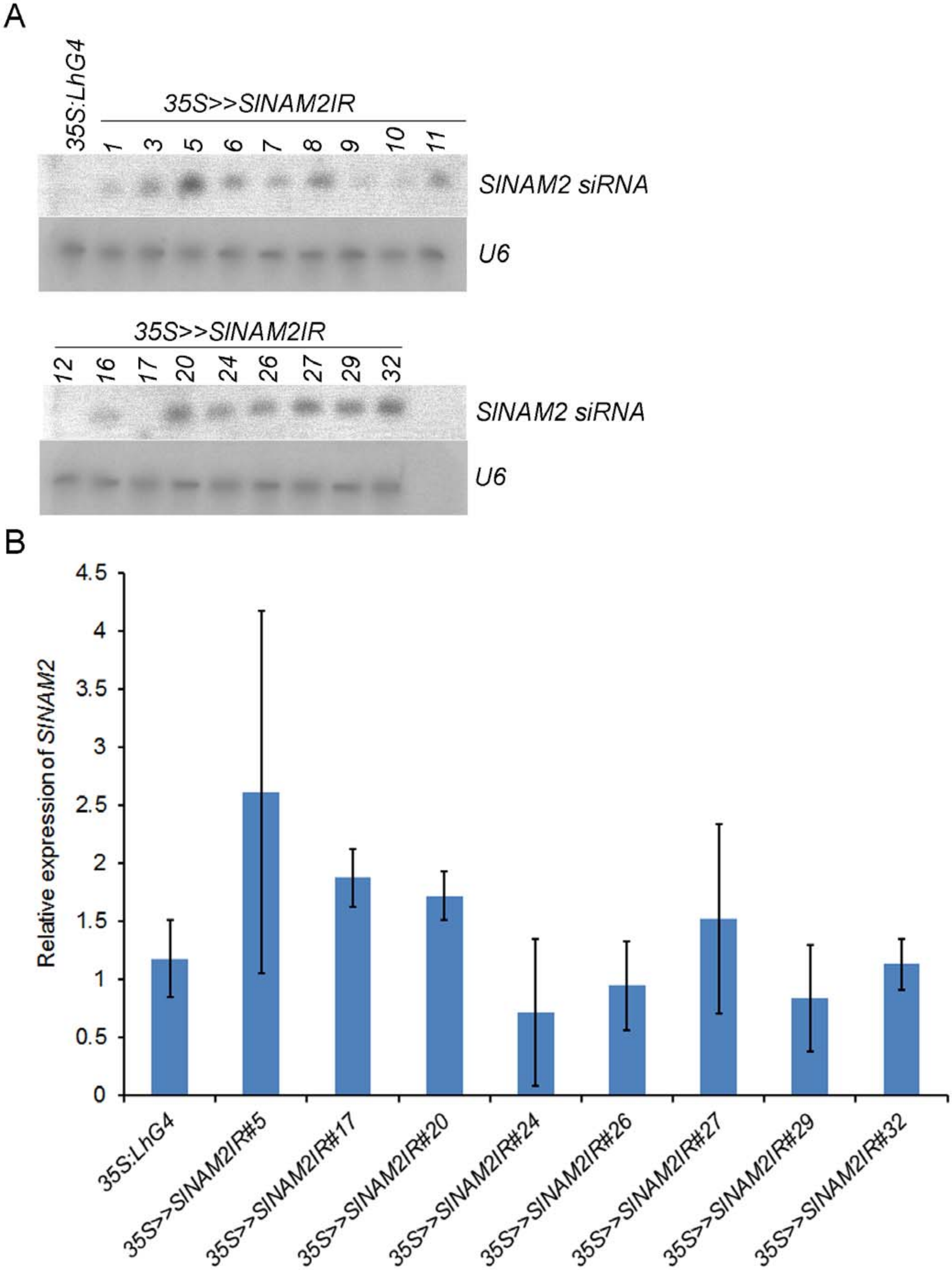
A
35S:LhG4
35S>>SlNAM2IR
1 3 5 6 7 8 9 10 11
SlNAM2 siRNA
U6
35S>>SlNAM2IR
12 16 17 20 24 26 27 29 32
SlNAM2 siRNA
U6
B
Relative expression of SlNAM2
0
0.5
1
1.5
2
2.5
3
3.5
4
4.5
35S:LhG4
35S>>SlNAM2IR#5
35S>>SlNAM2IR#17
35S>>SlNAM2IR#20
35S>>SlNAM2IR#24
35S>>SlNAM2IR#26
35S>>SlNAM2IR#27
35S>>SlNAM2IR#29
35S>>SlNAM2IR#32

Figure S5
